# Going deeper with morphologically detailed neural networks by simulation-based gradient propagation

**DOI:** 10.3389/fncom.2026.1904220

**Published:** 2026-09-10

**Authors:** Gan He, Kai Du, Tiejun Huang

**Affiliations:** 1 National Key Laboratory for Multimedia Information Processing, School of Computer Science, Peking University, Beijing, China; 2 Beijing Academy of Artificial Intelligence, Beijing, China

**Keywords:** adversarial robustness, cable theory, dendritic computation, learning rule, multi-compartment model, synaptic plasticity

## Abstract

Morphologically detailed dendrites possess powerful computational capabilities but are computationally expensive. Therefore, exploring how large-scale, detailed multi-compartment neural networks achieve learning proves challenging, presenting significant obstacles both in learning algorithms and simulation infrastructures. Here, we provide an extension to the DeepDendrite framework to enable the construction and data-driven training of multi-layer, detailed multi-compartment neural networks with highly modularized layer components. The gradient at each layer is computed by simulating gradient-mirror neurons in the feedback pathway simultaneously with the detailed neurons in the feedforward pathway, providing a simulation-based implementation of backpropagation in detailed neural networks. We demonstrate comparable results on classic image classification datasets with fully-connected and convolutional architectures. Furthermore, we analyze transfer attack robustness between artificial neural networks (ANNs), detailed neural networks and single-compartment networks. In conclusion, we provide a useful framework to investigate learning in multi-layer detailed neural networks, potentially offering further insights into exploiting the computational potential of dendrites at a large scale.

## 1 Introduction

The success of deep learning in AI has been demonstrated across numerous fields (LeCun et al., 2015; Silver et al., 2016; Abramson et al., 2024; Guo et al., 2025), with its core technical pillars being deep network architectures (Krizhevsky et al., 2012; He et al., 2016; Vaswani et al., 2017) and the backpropagation algorithm (Rumelhart et al., 1986; Schmidhuber, 2015). However, unlike the point-like neurons (McCulloch and Pitts, 1943; Rosenblatt, 1958) utilized in mainstream deep learning models, real neurons in the brain possess elaborate dendrites that integrate synaptic inputs in complex, nonlinear ways. Both active nonlinear mechanisms and passive cable properties contribute to dendritic computation (Payeur et al., 2019; Poirazi and Papoutsi, 2020). Passive dendrites, through morphology-dependent filtering and signal attenuation, can also reshape neuronal input-output transformations (Rall, 1967; Koch et al., 1983; Stuart and Spruston, 1998). Therefore, single neurons can effectively function as multi-layer neural networks (Poirazi et al., 2003; Ujfalussy et al., 2018; Beniaguev et al., 2021). The mismatch between neuroscience and AI highlights a key ingredient of biological intelligence–dendritic computation (London and Häusser, 2005; Stuart and Spruston, 2015) –that is often ignored. Consequently, the joint application of deep networks and learning algorithms to biophysically detailed multi-compartment neurons remains largely unexplored.

One reason for this mismatch is that the high computational cost of realistic dendritic trees severely limits their application (Häusser and Mel, 2003; Moldwin and Segev, 2020; Bicknell and Häusser, 2021; Zhang et al., 2023). Significant impediments must be overcome both in investigating the synaptic learning rules that operate across network of dendrites and in enhancing simulation platforms to support high-performance training with multi-compartment models. Unlike deep learning, which relies on well-established frameworks such as TensorFlow and PyTorch (Abadi et al., 2016; Paszke et al., 2019), mainstream detailed neuronal simulators (Hines and Carnevale, 1997; Kumbhar et al., 2019) focus exclusively on simulation itself, giving little consideration to support large-scale training. For example, utilities to support batch training are missing, and neuronal models are frequently loaded and unloaded on computing devices when switching samples.

To address this issue, recent studies have focused on enhancing or redesigning detailed neuronal simulators to incorporate utilities for data-driven training, resulting in emerging simulation platforms such as DeepDendrite and JAXLEY (Zhang et al., 2023; Deistler et al., 2025). These platforms have demonstrated that detailed neuronal models can be optimized for data-driven tasks, highlighting task-constrained training of biophysical models as an emerging direction in computational neuroscience. However, utilities to support modular construction of deep networks are rather limited, and results have only been demonstrated for detailed neural networks with a single hidden layer (Wybo et al., 2023; Zhang et al., 2023; Deistler et al., 2025), leaving deep detailed networks unexplored.

**FIGURE 1:**
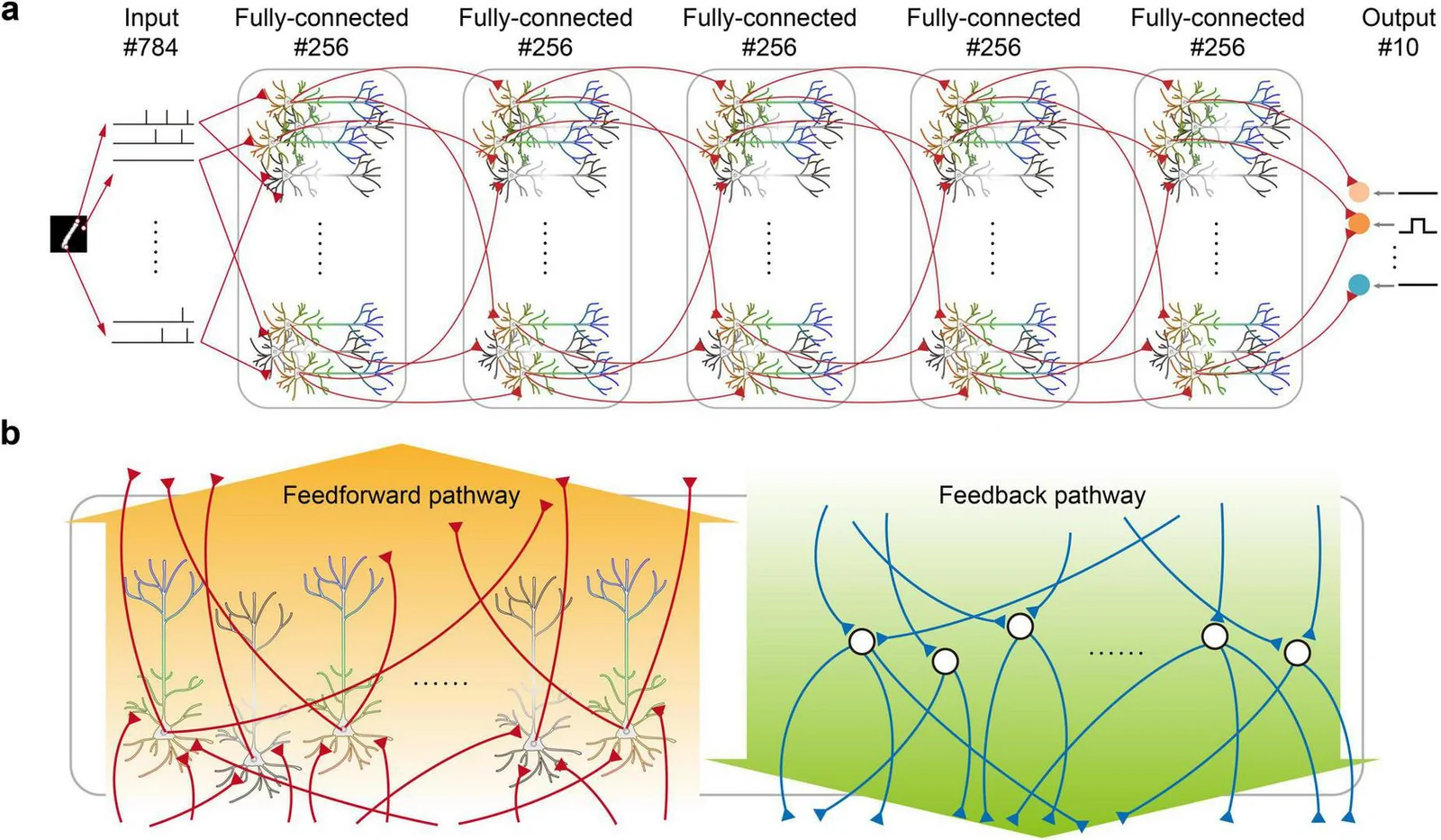
Multi-layer detailed neural network construction using modularized layers. **(a)** An example of a fully-connected detailed neural network for image classification. The pixel values of an image are converted into spike trains to serve as input to the network. The detailed neural network is constructed by stacking these highly modularized layers. Only the feedforward pathway is shown for clarity. **(b)** Diagram of a modularized layer. Each layer comprises detailed multi-compartment neurons in the feedforward pathway for inference, and single-compartment gradient-mirror neurons in the feedback pathway for error backpropagation. Both pathways are simulated simultaneously. The somatic gradients of the feedforward detailed neurons can be closely approximated by the somatic voltages of gradient-mirror neurons.

In this work, we extend the established DeepDendrite framework to support the construction and data-driven training of multi-layer, detailed multi-compartment neural networks. We provide highly modularized layer components (e.g., input, output, fully-connected, and convolutional layers) as basic building blocks for network construction (Figure 1a). Each layer comprises multi-compartment neurons in the feedforward pathway for inference and single-compartment gradient-mirror neurons in the feedback pathway for error backpropagation (Figure 1b). Simulating both pathways simultaneously, the somatic gradients of feedforward neurons are approximated via the somatic voltages of gradient-mirror neurons, providing a simulation-based implementation of error backpropagation in detailed neural networks.

We conduct experiments on classic image classification datasets using fully-connected and convolutional networks, demonstrating that our framework can achieve comparable results to artificial neural networks (ANNs) of identical architectures. Additionally, to investigate potential computational advantages brought by dendrites, we target the networks with transfer adversarial attacks (Goodfellow et al., 2015; Papernot et al., 2016). The results show that fully-connected detailed neural networks consistently outperform ANNs under high attack strength. Furthermore, we compare transfer adversarial robustness between single-compartment and detailed fully-connected networks, demonstrating that dendritic compartments provide an additional contribution to this effect. In conclusion, we provide a useful framework to advance explorations in training multi-layer detailed neural networks, potentially offering new possibilities into investigating how dendritic structure influence task-driven neural computation.

## 2 Materials and methods

### 2.1 Code availability statement

The source code for the modified DeepDendrite and the modularized layer APIs is available at https://github.com/MS-GEB/DeepDendrite-modularization.

### 2.2 Detailed neurons in the feedforward pathway

We utilize morphologically detailed multi-compartment models with passive dendrites and active synapses for inference in the feedforward pathway, following the HPC-Net model proposed in DeepDendrite (Zhang et al., 2023). Although active ion channels are not incorporated in the model to preserve mathematical tractability, passive dendrites are computationally important because morphology imposes location-dependent filtering, attenuation, and transfer-resistance structure on synaptic integration (Rall, 1967, 1994; Rinzel and Rall, 1974). Here, we provide a detailed description of the theory behind our approach, which was undisclosed in the original DeepDendrite paper. For passive dendrites, the voltage change at any location in response to arbitrary current inputs can be explicitly and accurately expressed following transfer impedance theory (Butz and Cowan, 1974; Koch et al., 1983; Koch and Poggio, 1985) as:


$$\begin{array}{c} V_{k}\,\left(t\right)=\overset{N}{\underset{j=1}{\sum }}K_{k\,j}\,\left(t\right)*I_{j}\,\left(t\right)=\overset{N}{\underset{j=1}{\sum }}\int_{0}^{t}K_{k\,j}\,\left(t-s\right)\,I_{j}\,\left(s\right)\,ds \end{array}$$

where *V*_*k*_ (*t*) is the relative change of voltage at location *k* relative to resting potential, *N* is the number of current inputs, *K*_*kj*_ (*t*) is the impulse response or Green’s function between current input location *j* and voltage output location *k* over elapsed time *t*, and * denotes convolution. For current inputs with a time-invariant mean, i.e., $𝔼\,\left(I_{j}\,\left(t\right)\right)={\bar{I}}_{j}$, the steady-state expected voltages can be computed by:


$$\begin{array}{c} 𝔼\,\left(V_{k}\,\left(\infty \right)\right)=\overset{N}{\underset{j=1}{\sum }}{\bar{I}}_{j}\,\int_{0}^{\infty }K_{k\,j}\,\left(t-s\right)\,ds=\overset{N}{\underset{j=1}{\sum }}{\bar{I}}_{j}\,R_{k\,j} \end{array}$$

where *R*_*kj*_ is the transfer resistance between location *j* and *k* on the dendrites, defined as $R_{k\,j}:=\int_{0}^{\infty }K_{k\,j}\left(t-s\right)ds$. The neurons in different layers are connected by synapses with active conductances, where the postsynaptic current *I*_*j*_ (*t*) depends nonlinearly on the somatic voltage of the presynaptic neuron *j* in the previous layer, *V*_in,*j*_ (*t*):


$$I_{j}\,\left(t\right)=w_{j}\,g_{j}\,f\,\left(V_{\text{in},j}\,\left(t\right)\right)$$

where *w_j_* is the synaptic weight, *g_j_* is the normalization factor, and *f*(⋅) is the nonlinear activation function. As we are primarily interested in the somatic voltages of each neuron, we omit subscript *k* and add subscript *i* to denote the somatic voltage of neuron *i* as *V*_*i*_ (*t*), and the transfer resistance between dendritic location *j* and the soma of neuron *i* as *R*_*ij*_. Assuming *V*_in,*j*_ (*t*) fluctuates slightly around its time-invariant mean ${\bar{V}}_{\text{in},j}$, we can combine the above equations to demonstrate that the expected somatic voltages of neurons in the next layer are also constant at steady state:


$${\bar{V}}_{i}:=𝔼\left(V_{i}\left(\infty \right)\right)\begin{array}{c} =\overset{N_{i}}{\underset{j=1}{\sum }}w_{i\,j}\,g_{i\,j}\,R_{i\,j}\,f\,\left({\bar{V}}_{\text{in},j}\right) \end{array}$$

Therefore, we can describe the computation in our multi-layer detailed neural networks in terms of steady-state expected voltages as:


$${\bar{V}}_{l+1}=W_{l+1}\,f\,\left({\bar{V}}_{l}\right)$$

where ${\bar{V}}_{l}$ is the expected somatic voltage vector of neurons in layer *l*, and *W*_*l* + 1_ is the matrix of equivalent connection weights between layer *l* and *l* + 1, defined as *W*_*l* + 1, *ij*_ = *w*_*l* + 1, *ij*_*g*_*l* + 1, *ij*_*R*_*l* + 1, *ij*_. The resulting formula regarding steady-state expected voltages shares a similar mathematical form with conventional ANNs, providing a theoretical basis for well-established ANN learning methods (e.g., backpropagation) to be readily transferred to multi-layer detailed neural networks.

### 2.3 Gradient-mirror neurons in the feedback pathway

We define gradient-mirror neurons as the neurons utilized in the feedback pathway whose voltages represent gradient signals of the feedforward pathway. It should be noted that the term “gradient-mirror neuron” is unrelated to action-observation mirror neurons (Rizzolatti and Craighero, 2004; Iacoboni, 2009). The design of simulating single-compartment gradient-mirror neurons for error backpropagation stems from two considerations. First, from a computational perspective, using a unified simulation substrate for inference and optimization provides a convenient way to construct and train detailed neural networks. Second, to enable high-performance training, we seek to unify the computation of simulation and backpropagation so that learning is not interrupted by alternating between different computing mechanisms.

As a result of this design, the somatic gradients of detailed neurons in the feedforward pathway can be approximated by the simulated somatic voltages of gradient-mirror neurons in the feedback pathway. This unifies gradient computation with simulation and provides a simulation-based implementation of backpropagation. Specifically, the backpropagation rule for somatic gradients is:


∂⁡L∂⁡V¯l,j=∑i=1Nl+1∂⁡L∂⁡V¯l+1,iwl+1,i⁢jgl+1,i⁢jRl+1,i⁢jf(V¯l,j)′

where *L* is the loss function at the output layer. The synaptic input from gradient-mirror neuron *i* in layer *l* +1 to gradient-mirror neuron *j* in layer *l* is defined as:


$$I_{l+1,j\,i}^{\text{mirror}}=g_{\text{m}}\,\frac{\partial L}{\partial {\bar{V}}_{l+1,i}}\,w_{l+1,i\,j}\,g_{l+1,i\,j}\,R_{l+1,i\,j}\,f^{\prime }\,\left({\bar{V}}_{l,j}\right)$$

where *g*_*m*_ is the synaptic conductance. The steady-state expected voltages of gradient-mirror neurons are then given by:


$${\bar{V}}_{l,j}^{\text{mirror}}=\overset{N_{l+1}}{\underset{i=1}{\sum }}r_{\text{m}}\,I_{l+1,j\,i}^{\text{mirror}}$$

where *r*_*m*_ is the input resistance of the single-compartment gradient-mirror neuron. By setting *g*_m_ = 1/*r*_m_ and combining the above equations, we obtain ${\bar{V}}_{l,j}^{\text{mirror}}=\partial L/{\bar{V}}_{l,j}$, demonstrating that the somatic gradients of feedforward neurons match the expected voltages of the gradient-mirror neurons at steady state. This approach should therefore be regarded as a computational mechanism for gradient propagation in detailed neural simulations rather than a biologically local learning rule.

### 2.4 Synaptic plasticity rule

The original synaptic plasticity rule in DeepDendrite computes the gradient at each time step and uses the averaged gradient to guide learning. However, when applied to multi-layer networks, we found such a setting occasionally leads to unstable results due to rapid voltage variations. To stabilize training, we compute weight updates from expected voltages:


$$△\,w_{l,i\,j}=-\eta \,\frac{\partial L}{\partial w_{l,i\,j}}=-\eta \,\frac{\partial L}{\partial {\bar{V}}_{l,i}}\,g_{l,i\,j}\,R_{l,i\,j}\,f\,\left({\bar{V}}_{l-1,j}\right)$$

where η is the learning rate. In practice, we use running averages to approximate expected voltages in an online manner.

### 2.5 Input patterns

We adopt image classification tasks as typical benchmarks for data-driven learning. To convert a static image into spike train inputs for neuronal simulation, we follow the method in DeepDendrite to convert normalized pixel values into the onsets and intervals of homogeneous spike trains, ensuring that the postsynaptic potentials maintain constant expectations at steady state. Specifically, for each grayscale or color channel value of a pixel normalized between 0 and 1, denoted by *p*(*x*, *y*, *c*) [where (*x*, *y*) is the pixel location on image and *c* is the channel index], the corresponding spike train has a constant interspike interval of:


$$\begin{array}{c} I\,S\,I\,\left(x,y,c\right)=\frac{I\,S\,I_{\text{min}}}{p\,\left(x,y,c\right)+0.01} \end{array}$$

in milliseconds, where *ISI*_min_ is the minimum interval when *p*(*x*, *y*, *c*) reaches its maximum value. The onset of the first spike is given by *ISI*(*x*, *y*, *c*) + *T*_on_, where *T*_on_ is a constant parameter. The simulation time per image is denoted by *T*. In the original setting of DeepDendrite, *ISI*_min_ = 5ms, *T*_on_ = 9ms, and *T* = 50ms. However, in multi-layer networks, we found this duration insufficient to drive responses in deeper layers. Therefore, we extended the simulation time per image to *T* = 500ms, and the period for learning is set between 200ms and 500ms for running average computation. The input spikes are received by synapses with single-exponential conductance, with a decay time constant of 5ms and a reversal potential of 1mV.

## 3 Results

### 3.1 Gradient-mirror neurons enable effective training of multi-layer detailed neural networks

We demonstrate the learning capabilities of our proposed framework on the MNIST (LeCun et al., 1998) and CIFAR-10 (Krizhevsky, 2009) image classification datasets using typical fully-connected and convolutional architectures following Bartunov et al. (2018). We chose not to experiment with larger networks or more challenging datasets, such as ImageNet (Deng et al., 2009), due to excessive training times. Even when utilizing DeepDendrite on a single NVIDIA A100 GPU, we estimate that completing 60 epochs of training for a convolutional network on CIFAR-10 would take 4 weeks, and a single epoch of training on ImageNet would require a month. As a result, we focus our experiments on fully-connected and convolutional networks on MNIST, and a fully-connected network on CIFAR-10, which require 1 day, 3 days, and 1 week, respectively, to complete 60 epochs of training. For each network we performed multiple independent runs with different random seeds (*N* = 3). For the convolutional network on MNIST, the feedforward neurons in the convolutional layers are single-compartment, and the simulation time step is enlarged to 10ms to reduce training time to an affordable amount.

The detailed multi-compartment neurons in the feedforward pathway were constructed using the morphology of a layer 2/3 pyramidal neuron from the mouse brain [Allen Institute for Brain Science (2015); ID 472299294]. We retained the passive dendritic properties of the model and set the reversal potential to 0 mV, following the practice of HPC-Net in DeepDendrite. For a fair comparison with ANN baselines, we utilized identical network architectures, activation functions, number of synaptic connections between neurons, weight initializers (He et al., 2015), batch sizes, loss functions, optimizers, learning rates, and training epochs, as summarized in Table 1. We employed cross-entropy as the loss function, stochastic gradient descent (SGD) without momentum or weight decay as the optimizer, and refrained from applying techniques like data augmentation or normalization.

**TABLE 1:** Experiment specification of detailed neural networks and ANNs.

Configuration	MNIST		CIFAR-10	ANNs
	Fully-connected	Convolutional	Fully-connected	
Architecture	FC 256 FC 256 FC 256 FC 256 FC 256 Softmax 10	(3 × 3, 32, 2) (3 × 3, 64, 2) FC 1024 Softmax 10	FC 1024 FC 1024 FC 1024 Softmax 10	Same as corresponding detailed neural networks
Platform	DeepDendrite			PyTorch
Simulation time step (ms)	5	10	5	N/A
Input spike interval (ms)	50/(p(x, y, c) + 0.01)			N/A
Input spike start (ms)	90 + 50/(p(x, y, c) + 0.01)			N/A
Activation function	ReLU			
Synaptic connections between neurons	1			
Weight initializer	Kaiming uniform			
Batch size	16	2	8	Same as corresponding detailed neural networks
Optimizer	SGD without momentum or weight decay			
Learning rate	3e-2	1e-2	5e-4	Same as corresponding detailed neural networks
Training epochs	60			

The format for locally-connected layers is (kernel size, number of output channels, stride).

The learning rates were first selected for the ANNs to yield the highest clean test accuracy, and the identical rates were subsequently applied to train the detailed neural networks. The final performances of the detailed neural networks and corresponding ANNs are summarized in Table 2. Our results show that detailed neural networks can achieve clean test accuracies comparable to their ANN counterparts, indicating that the gradient-mirror neurons in our framework can effectively leverage voltages to propagate gradients backward through layers and enable robust training of multi-layer detailed neural networks.

**TABLE 2:** Comparison of clean test accuracy (%) between multi-layer detailed neural networks and corresponding ANNs (*N* = 3).

	MNIST		CIFAR-10
Network type	Fully-connected	Convolutional	Fully-connected
Deep detailed network	98.42 ± 0.05	98.96 ± 0.10	52.63 ± 0.14
ANN	98.45 ± 0.08	99.06 ± 0.06	52.95 ± 0.27

### 3.2 Enhanced transfer attack robustness in detailed neural networks

We next investigate possible computational advantages brought by dendrites. In the original DeepDendrite study, we demonstrated increased resilience to transfer adversarial attacks in the HPC-Net, but the network comprised only one hidden layer of detailed dendritic neurons and evaluations were restricted to MNIST and Fashion-MNIST (Xiao et al., 2017). To verify whether this conclusion scales to larger networks and datasets, we performed transfer attacks on our trained detailed neural networks. Notably, because the onsets and intervals of spike trains converted from an image are fixed, no noise is added during the training of detailed neural networks. The adversarial samples were obtained from a third network with a significantly different architecture–a 20-layer Residual Neural Network (ResNet) (He et al., 2016) –to ensure a fair comparison of transfer attack robustness. We utilized Foolbox (Rauber et al., 2017, 2020) alongside the Fast Gradient Sign Method (FGSM) (Goodfellow et al., 2015) to generate adversarial noise.

**FIGURE 2:**
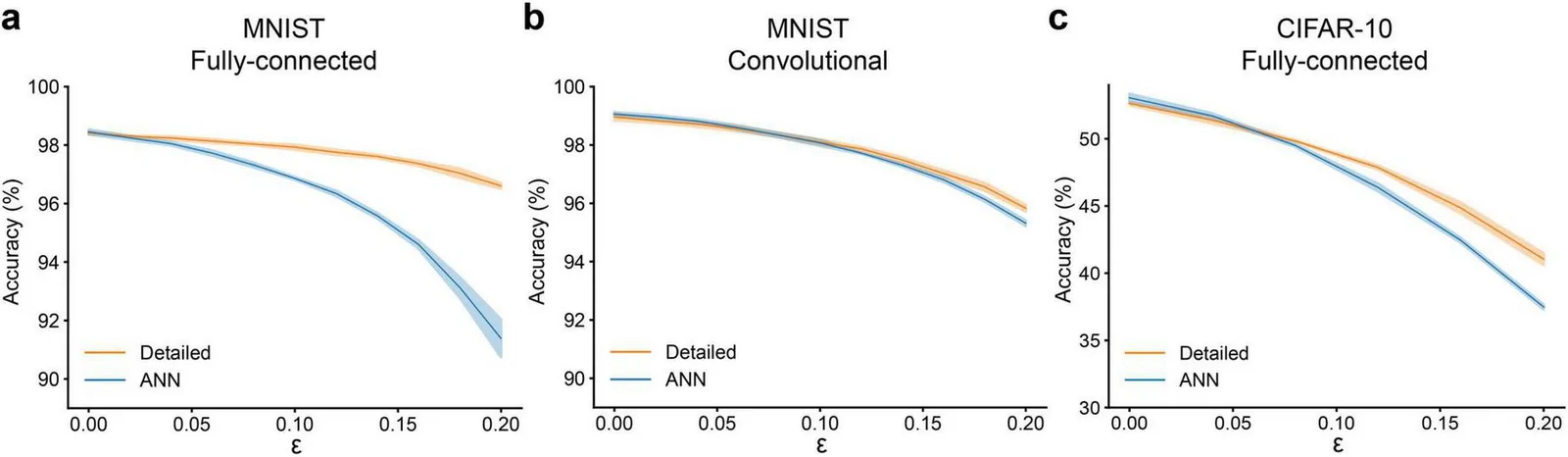
Comparison of FGSM transfer attack robustness between detailed neural networks and corresponding ANNs. *N* = 3. **(a)** Fully-connected network evaluated on MNIST. **(b)** Convolutional network evaluated on MNIST. **(c)** Fully-connected network evaluated on CIFAR-10.

The results for the three network architectures on MNIST and CIFAR-10 are illustrated in Figure 2. For fully-connected architectures, the detailed neural networks achieved average improvements of 5.22% on MNIST and 3.56% on CIFAR-10 over ANNs when attack strength reached ε = 0.2. For the convolutional architecture, the performance difference was less pronounced, with the detailed network slightly exceeding the ANN by an average of 0.51% on MNIST. We argue that this marginal improvement is likely because the convolutional architectures already provided sufficient transfer adversarial robustness on this dataset (at ε = 0.2, an average of 95.32% for the convolutional ANN, compared with 91.39% for the fully-connected ANN). Furthermore, the use of point neurons instead of dendritic neurons in the convolutional layers, alongside the enlarged simulation time step, may have impaired the performance on detailed neural networks.

To obtain a more comprehensive comparison of the fully-connected networks, we expanded our experiments with an additional base model for adversarial sample generation and incorporated alternative attack methods. We trained an 11-layer VGG network (Simonyan and Zisserman, 2015) as a base model, which achieved a test accuracy of 99.53% on MNIST and 84.18% on CIFAR-10. To diversify the attack methods, we adopted a 5-step Projected Gradient Descent (PGD) (Madry et al., 2018) and DeepFool (Moosavi-Dezfooli et al., 2016) in addition to FGSM. Consequently, we evaluated 5 additional combinations of transfer attack samples for both MNIST and CIFAR-10.

**FIGURE 3:**
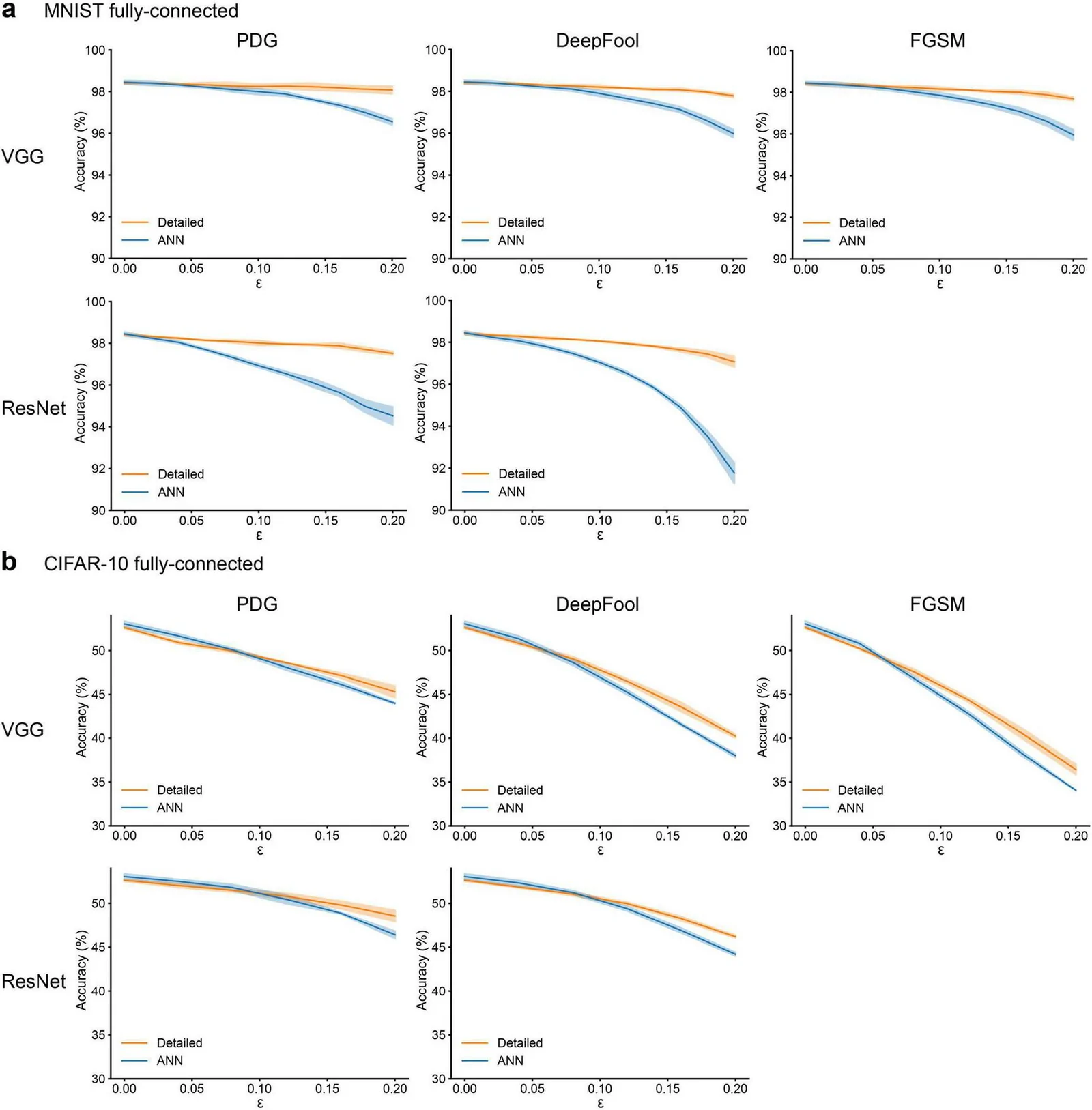
Transfer attack robustness comparison between fully-connected detailed neural networks and corresponding ANNs across combinations of base models and attack methods. Results for the ResNet+FGSM configuration are shown in Figure 2. *N* = 3. **(a)** Comparison on MNIST. **(b)** Comparison on CIFAR-10.

The prediction accuracies of the fully-connected detailed neural networks and ANNs on these samples are illustrated in Figure 3. Our results demonstrate that the detailed neural networks consistently outperformed their ANN counterparts across all combinations under high attack strength. For the more successful attack configurations, detailed neural networks achieved an average improvement of 5.30% for MNIST and 2.38% for CIFAR-10 when the attack strength reached ε = 0.2 (e.g., DeepFool on ResNet for MNIST; FGSM on VGG for CIFAR-10). These results indicate that fully-connected detailed neural networks trained with our framework are endowed with enhanced transfer adversarial robustness under high attack strength.

### 3.3 Dendrites provide an additional contribution to transfer adversarial robustness

The improved transfer adversarial robustness in detailed neural networks might be attributed to multiple factors including dendritic morphology, spike-train input encoding, temporal averaging and others. To further assess the contribution of dendrites, we performed ablation experiments on fully-connected single-compartment networks that composed only the somatic compartments in detailed neuron models. The same experiment specifications were applied as in the detailed neural networks for training and evaluation, including the same spike encoding and temporal averaging.

**FIGURE 4:**
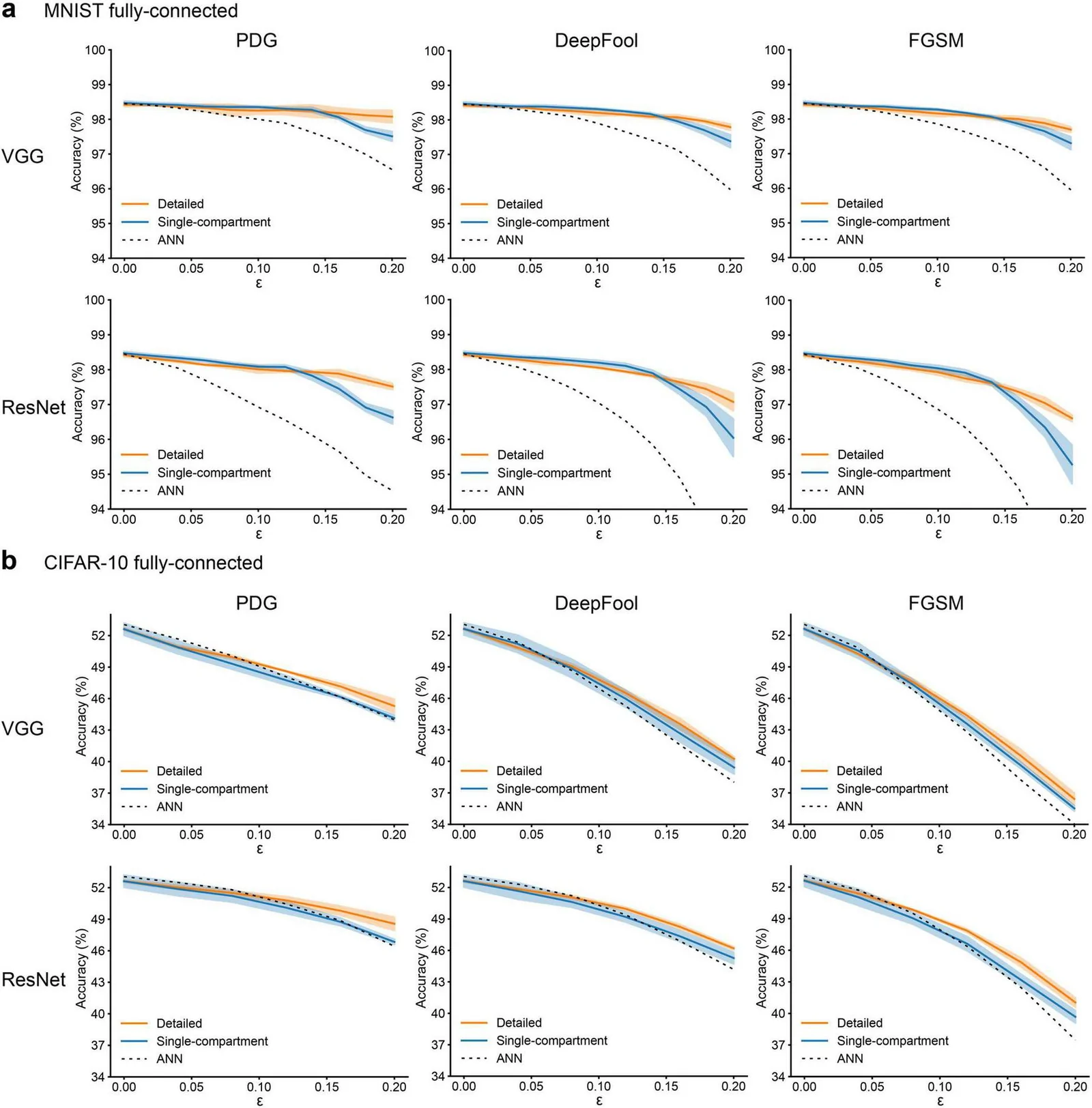
Transfer attack robustness comparison between fully-connected detailed neural networks and corresponding single-compartment networks. The single-compartment networks preserve the same spike encoding and temporal integration scheme as the detailed networks, while removing dendritic compartments. ANN accuracy means are shown as baselines. *N* = 3. **(a)** Comparison on MNIST. **(b)** Comparison on CIFAR-10.

On both datasets, the clean test accuracy for single-compartment networks was largely in line with the detailed networks, achieving an average of 98.47% ± 0.10% on MNIST and 52.60% ± 0.57% on CIFAR-10. We then targeted the single-compartment networks with transfer adversarial samples from all 6 combinations evaluated in the previous experiment; the prediction accuracies are illustrated in Figure 4. On MNIST, single-compartment networks maintained similar accuracies to detailed networks under low attack strength. Under high attack strength, however, the performance of single-compartment networks quickly dropped, and detailed networks outperformed their single-compartment counterparts across all combinations (at ε = 0.2, min: 0.39% for FGSM on VGG, max: 1.33% for FGSM on ResNet). Additionally, single-compartment networks constantly outperformed ANNs, indicating that factors other than dendrites also contribute to the model’s transfer adversarial robustness. On CIFAR-10, the detailed networks also maintained a consistent average accuracy improvement over single-compartment networks across all combinations under high attack strength (at ε = 0.2, min: 0.82% for DeepFool on VGG, max: 1.73% for PGD on ResNet). Our results indicate that dendritic compartments provide an additional contribution to the transfer attack robustness of fully-connected detailed neural networks under high attack strength.

## 4 Discussion

Biophysically detailed multi-compartment models are powerful tools to decipher the computational principles of the brain. However, the application of learning in deep detailed networks remains largely unexplored due to excessive computational cost that poses significant challenges both in developing learning algorithms and advancing simulation platforms. In this work, we build upon the established DeepDendrite framework to provide an extension for constructing and training multi-layer detailed neural networks. By using gradient-mirror neurons to backpropagate errors via simulation, we provide a simulation-based implementation of backpropagation in detailed neural networks. Utilizing this framework, we achieved learning performance comparable to ANNs of identical architectures on MNIST and CIFAR-10. The fully-connected detailed neural networks exhibited improved performance under high-strength transfer attacks, and further ablation experiments suggested that dendritic compartments provide an additional contribution to this effect.

Although we construct detailed networks with architectures similar to ANNs and employ backpropagation as a mathematical basis for learning, our primary goal is not to replicate the success of ANNs. Instead, we seek to investigate the feasibility of training detailed networks at scale to better resemble the cognitive systems of the brain. To this end, two important aspects are considered. First, we build our extension upon the advanced DeepDendrite platform, which employs the computationally optimal and accurate method of Dendritic Hierarchical Scheduling (DHS) to simulate dendrites and is optimized for GPU hardware. Therefore, our framework serves as an excellent benchmark to assess the computational time and resources required to train large-scale detailed networks on data-driven tasks. Second, we implement the computations required for learning directly via neuronal simulation. This provides a unified computational framework in which neuronal dynamics and optimization signals are represented within the same simulation environment.

It is worth noting that the observed transfer adversarial robustness in fully-connected detailed neural networks likely stems from a combination of factors, including dendritic morphology, spike-train input encoding, temporal averaging, conductance normalization, activation scaling, and other simulation-specific details. Our ablation experiments against single-compartment networks, which controlled for encoding and temporal averaging, suggest that dendrites provide an additional contribution to transfer adversarial robustness under high attack strength. However, the relative contributions of these factors warrant systematic investigation in future studies.

We also emphasize that our conclusions regarding adversarial robustness are specifically confined to the transfer attack setting, where adversarial samples are generated from an external source model with a significantly different architecture. A lower transferability of adversarial perturbations could arise from various factors, including differences in input encoding, temporal dynamics, or gradient mismatch between the source model and the detailed network, rather than from intrinsic robustness in the white-box or adaptive sense. We do not claim that detailed neural networks are inherently robust against adaptive attacks that incorporate the image-to-spike encoding and network simulation into the attack pipeline. Such white-box attacks would be a valuable direction for future work.

Additionally, the gradient-mirror neuron implementation in its current form relies on precise knowledge of feedforward weights, normalization factors, and dendritic transfer resistances to define feedback synaptic inputs. This means that, despite being a simulation-based implementation, our method still faces challenges related to the weight-transport problem when considered from a biological plausibility perspective. Addressing this limitation and incorporating more biologically realistic learning rules–such as those based on random feedback weights (Lillicrap et al., 2016) or local synaptic plasticity (Sacramento et al., 2018; Lillicrap et al., 2020; Payeur et al., 2021) –into our framework remains an important direction for future research.

We utilize detailed neurons with passive dendrites and active synapses in our current framework to preserve mathematical tractability. However, real neurons in the brain possess ion channels and synapses with diverse active properties, enabling dendrites to perform more complex computations (London and Häusser, 2005; Payeur et al., 2019; Poirazi and Papoutsi, 2020). Therefore, to build more realistic learning models of the brain, additional biological and cognitive principles must be incorporated into the framework, such as active dendrites, structured connectivity, neuromodulators, etc. Future research is needed to identify rules that can facilitate learning with the involvement of these principles and to explore their computational implications for large-scale learning models.

Although our current experiments were limited in scope and computational power, future efforts will be dedicated to developing a scalable framework that can effectively leverage multi-GPU systems, incorporating biological principles such as active dendrites, and extending evaluations across a wider range of tasks and domains. We explicitly acknowledge that multi-GPU support and further algorithmic innovations are essential for scaling to more challenging datasets and deeper architectures. We also note that our current results are limited to relatively small-scale architectures (fully-connected networks on MNIST and CIFAR-10, with convolutional experiments employing single-compartment neurons in the convolutional layers as a practical compromise to reduce computational cost). Extending to genuinely deep, all-dendritic convolutional networks on large-scale datasets such as ImageNet remains an important direction for future work. By overcoming these limitations, we can further advance our understanding of biophysically detailed neural networks and open up new opportunities for exploiting the potential of dendrites at a large scale.

## Data Availability

Publicly available datasets were analyzed in this study. The MNIST dataset used in this study is available at http://yann.lecun.com/exdb/mnist. The CIFAR-10 dataset used in this study is available at https://www.cs.toronto.edu/~kriz/cifar.html. The source code for the modified DeepDendrite and the modularized layer APIs is available at https://github.com/MS-GEB/DeepDendrite-modularization.
